# Prevalence of herbal medicine use for maternal conditions in Tanzania: a systematic review and meta-analysis

**DOI:** 10.3389/fphar.2025.1637891

**Published:** 2025-09-02

**Authors:** Hamisi S. Japhari, Susan F. Rumisha, Jackline D. Nkoma, Emanuel L. Peter

**Affiliations:** ^1^ Mabibo Traditional Medicine Research Center, National Institute for Medical Research, Dar Es Salaam, Tanzania; ^2^ Child Health Analytics, Malaria Atlas Project, Telethon Kids Institute, Nedlands, WA, Australia; ^3^ School of Population Health, Faculty of Health Sciences, Curtin University, Bentley, WA, Australia

**Keywords:** magnitude, herbal medicines, maternal conditions, prevalence, Tanzania

## Abstract

**Background:**

Studies reported the prevalence of herbal medicines used for various maternal conditions across regions in Tanzanian communities. However, the lack of a national estimate of herbal medicine use makes it challenging for policymakers, herbal medicine regulators, and healthcare practitioners to make informed decisions on herbal medicine-related policies and practices to optimize their contribution to maternal healthcare. This systematic review and meta-analysis synthesized the national prevalence of herbal medicine use for maternal conditions based on ethnomedical studies conducted in Tanzania.

**Methods:**

Authors systematically searched for published articles in PubMed, Embase, CINAHL, African Index Medicus, and Scopus databases from inception to 29 June 2025. Grey literature was obtained from Google, Google Scholar, OpenGrey, and ProQuest Dissertations & Theses. Articles published in the English language were retrieved. Later, two authors independently assessed the retrieved articles for eligibility and risk of bias using pre-determined criteria. We used the Cochran Q statistics and I^2^ tests to assess heterogeneity. Also, we applied the random-effects model to determine the pooled prevalence. Finally, subgroup and meta-regression analyses were performed to explore the source of heterogeneity.

**Results:**

About 22 studies with 5,248 women from 16 administrative regions of Tanzania were included in a narrative synthesis. These studies had a low to moderate risk of bias. Furthermore, fourteen studies (n = 4,817) were included in the meta-analysis. Overall, the average prevalence of herbal medicine use for maternal conditions was 46% [95%, CI: 34–58], I^2^ = 93.93%]. Similarly, the commonly managed maternal condition was labor induction 69% [95%, CI: 42–96], and its frequency of citation was (38%).

**Conclusion:**

The findings suggest that at least two of every five Tanzanian women are using herbal medicines. However, these findings could understate the national prevalence due to the inadequate availability of data from other regions. The prevalence of herbal medicine (46%) underscores the need for policymakers and healthcare practitioners to account for herbal medicine use while planning for maternal care. To achieve a robust generalizable estimate, data from better-designed ethnomedical surveys from all regions are still needed.

**Systematic Review Registration:**

https://www.crd.york.ac.uk/PROSPERO/view/CRD42023410082, identifier CRD42023410082.

## 1 Introduction

Maternal conditions include events occurring from conception to 42 days postpartum. Among these conditions, hemorrhage, sepsis, hypertensive disorders of pregnancy, obstructed labor, and unsafe abortion account for three-quarters of all maternal mortality in developing countries, particularly sub-Saharan Africa (Graham et al., 2006; Musarandega et al., 2021; Oyston and Baker, 2020) According to the 2022 Tanzania Demographic and Health Survey and Malaria Indicator Survey (TDHSMIS), the maternal mortality ratio (MMR) for Tanzania is 104 maternal deaths per 100,000 live births (MoH, 2023). Although the 2022 MMR estimate represents a significant decline from 432 deaths per 100,000 live births in 2012, it remains high, above the agreed sustainable development goal (SDG) target of reducing the MMR to 70 per 100,000 live births by 2030 (Tanzania National Bureau of Statistics, 2013). To sustain current gain in MMR reduction and accelerate efforts toward realizing the SDG target, several measures are being implemented, including strengthening implementing policies and strategies that are more targeted to vulnerable population groups across the country, decentralizing lifesaving comprehensive emergency obstetric care to health centers, strengthening referral systems, and involvement of public-private partnerships (PPP) to ensure a continuum of care, to name just a few (Prasad et al., 2022; Shija et al., 2011).

In the past decade, studies and anecdotal evidence indicated that Tanzanian women are increasingly using traditional medicine as a complementary or alternative healthcare service to manage various maternal conditions. For instance, Dika et al., 2017 reported about a quarter of the pregnant women delivering in tertiary referral hospitals in Mwanza city were using herbal medicines (HMs) as one form of traditional medicine (Dika et al., 2017). Similarly, the prevalence of HMs use during breastfeeding was reported by one out of every two women in the Morogoro region (Millinga et al., 2022). A similar high prevalence of HMs use at 61.2% was reported in the Tabora region (Kessy and Msalale, 2020a). These variations in HMs use across regions in Tanzania pose significant challenges among policymakers, regulators, and healthcare practitioners in designing HMs-related policies and practices aimed at optimizing its contribution to maternal healthcare.

The use of HMs among women for maternal conditions is linked to several factors, such as social status, ethnicity, and cultural traditions. Moreover, the extent of use for the wellbeing of either the mother or the unborn child varies across regions (Illamola et al., 2020). Furthermore, previous studies reported that prevention of incidents of nausea and vomiting, improving abdominal muscle tone and building stamina during labor and delivery, improving the health of the woman, ensuring positive pregnancy outcomes, and easing labor are among the determinants of HMs use (Attah et al., 2012; Felisian et al., 2022; Shewamene et al., 2017). Although studies indicated that the majority of women use HMs for various healthcare needs, there is no accurate national estimate on the prevalence of HMs use for maternal conditions among women in Tanzania. Therefore, we conducted this systematic review to answer the following research questions: (1) what is the average prevalence of HMs use for maternal conditions in Tanzania? (2) what are the major maternal conditions commonly managed by HMs among Tanzanian women? The findings of this review enhanced awareness of the extent to which women of reproductive age use HMs. Bridging this knowledge gap could potentially influence reproductive and child health programs to account for traditional medicine use while planning for interventions to improve maternal health. The improved maternal care outcomes in the form of identifying and addressing potential risks for HMs-conventional medicine interaction could contribute towards accelerating maternal mortality reduction. This reduction is critical in achieving SDG targets.

## 2 Methods

### 2.1 Protocol and registration

The systematic review and meta-analysis protocol were developed according to the preferred reporting items for systematic reviews and meta-analyses protocols (PRISMA-P) and registered with the Prospero database under registration number CRD42023410082, available from https://www.crd.york.ac.uk/PROSPERO/view/CRD42023410082 (Moher et al., 2015). Then, we reported our results following the PRISMA updated guidelines and the abstract checklist findings (Page et al., 2021).

### 2.2 Eligibility criteria

Published studies that reported the prevalence of HMs used for managing maternal conditions with or without other medications, and those that reported factors and/or indications for the use of HMs among Tanzanian women were included. The included studies were those with outcomes of interest, observational designs with ethics approval, and those published in English. To enhance the power of this review, relevant grey literature with methodological rigor, transparency, and reproducibility was also included. Articles with study designs, such as case-control, clinical trials, and case studies, were excluded because they do not report prevalence estimates. Furthermore, policy briefs, reviews, and those without full texts were excluded.

### 2.3 Information source and search strategy

The review team searched the following electronic databases from inception to 29 June 2025: Medline (PubMed), Embase, African Index Medicus, CINAHL, and Scopus. Grey literature, including preprints, conference papers, theses, and dissertations, was obtained from Google, Google Scholar, OpenGrey, and ProQuest Dissertations & Theses. The reference lists of included studies were screened for additional eligible studies not found through the search. The searches were re-run just before the final analysis to include the most recent eligible studies. Keywords and MeSH terms were used to create an effective search strategy. The strategy included the combination of keywords such as (Prevalence OR Magnitude OR Use OR Percent OR Trend) AND (“Herbal medicine” OR “Plant extracts” OR Herb OR “Traditional medicine” OR “Herbal remedies” OR “Medicinal plants”) AND (Tanzania), as detailed in Supplementary File 1.

### 2.4 Record management and selection

Identified articles were pooled into the Mendeley reference manager var. 2.1 (Elsevier) for duplicate removal and title and abstract screening. Then, two review authors (HSJ and ELP) independently screened the titles and abstracts of the identified studies for relevance to the review question. Two review authors (HSJ and JDN) assessed the full text of these potentially eligible studies for eligibility. Disagreements between authors over the eligibility of a particular study were resolved through consensus.

### 2.5 Data extraction and coding

Two review authors (HSJ and JDN) independently extracted the data using EpiData Manager var.4.6.0.6 software (Odense, Denmark). Before the data were extracted, the data extraction tool was pilot-tested with ten studies. Following the findings of the pilot test, improvements to the data extraction tool were made after reaching a consensus with co-authors. The standardized form was used to capture study characteristics, including age, region, indication for the use of HMs (type of maternal conditions treated), type of maternal period, factors associated with HMs use, study design, sample size, and the prevalence of HMs used for maternal conditions. Reviewers contacted the corresponding authors via email to obtain the full text of the identified study that had a missing full text. Corresponding authors who did not respond to our emails after three reminders at an interval of 2 months, such incomplete articles were excluded from our systematic review.

### 2.6 Risk of bias assessment

Two independent reviewers (HSJ and JDN) assessed the included articles for methodological validity and rigor prior to inclusion in the review using a standardized critical appraisal tool (i.e., the Joanna Briggs Institute Critical Appraisal Tool) (see Supplementary File 2). The risk of bias was classified as low (<50%), moderate (50%–79%), and high (≥80%) across assessed domains based on the proportion of checklist items met. Any disagreements that arose between the reviewers were resolved through consensus or with a third reviewer (ELP).

### 2.7 Strategy for data synthesis

#### 2.7.1 Qualitative synthesis

The narrative analysis method was used to summarize factors of HMs use among women for managing maternal conditions. The included studies were grouped based on factors influencing the use of HMs. The grouping was also based on the direction of influence. For each group, a narrative description was given (Rodgers et al., 2009). The direction of influence was determined by interpreting the context and language provided in the included studies. Specifically, factors were categorized as either promoting the use of HMs or not based on how they were described in the original study narratives. For example, when a study reported that “women are strongly holding to the traditional beliefs and local knowledge concerning the use of HMs for managing maternal conditions, and also affirmed that HMs are better than hospital drugs,” this was classified as a factor promoting HMs use, since the description reflected a positive inclination toward traditional practices over biomedical care.

#### 2.7.2 Quantitative synthesis of primary outcome

Meta-analysis was conducted to obtain the overall pooled estimate of the prevalence of HMs use for maternal conditions. The weighted prevalence estimates and their 95% confidence intervals (CI’s) were then calculated. The heterogeneity severity of the studies was assessed using the I^2^ test statistic. The I^2^ of 75% or more was considered as indicative of substantial heterogeneity (Borenstein et al., 2010; Deeks et al., 2008). The random effect model was applied to combine prevalence estimates from eligible studies because of the anticipated higher heterogeneity between included studies. Furthermore, the trend in HMs use was determined by comparing two time periods, i.e., the earlier 7-year period (2009–2016) and the later years (2017–2024); this stratification also considered adequate availability of studies in each period. To validate the model, we considered several approaches, including subgroup analysis by time period and year of publication. Furthermore, sensitivity analysis was explored by considering outliers, and finally, meta-regression analyses were performed to explore the sources of heterogeneity. Publication bias was assessed by testing the asymmetry of the funnel plot using Egger’s test (Begg and Mazumdar, 1994; Egger et al., 1997). The choropleth map was established to display the distribution pattern of the prevalence of HMs used for managing maternal conditions across regions in Tanzania.

This analysis was conducted in STATA version 17 (StataCorp LP, College Station, TX, 77845, United States), and the map was generated using ArcGIS Pro version 3.2 (https://www.esri.com/en-us/arcgis/products/arcgis-pro/overview).

## 3 Results

### 3.1 Study selection flow

**FIGURE 1:**
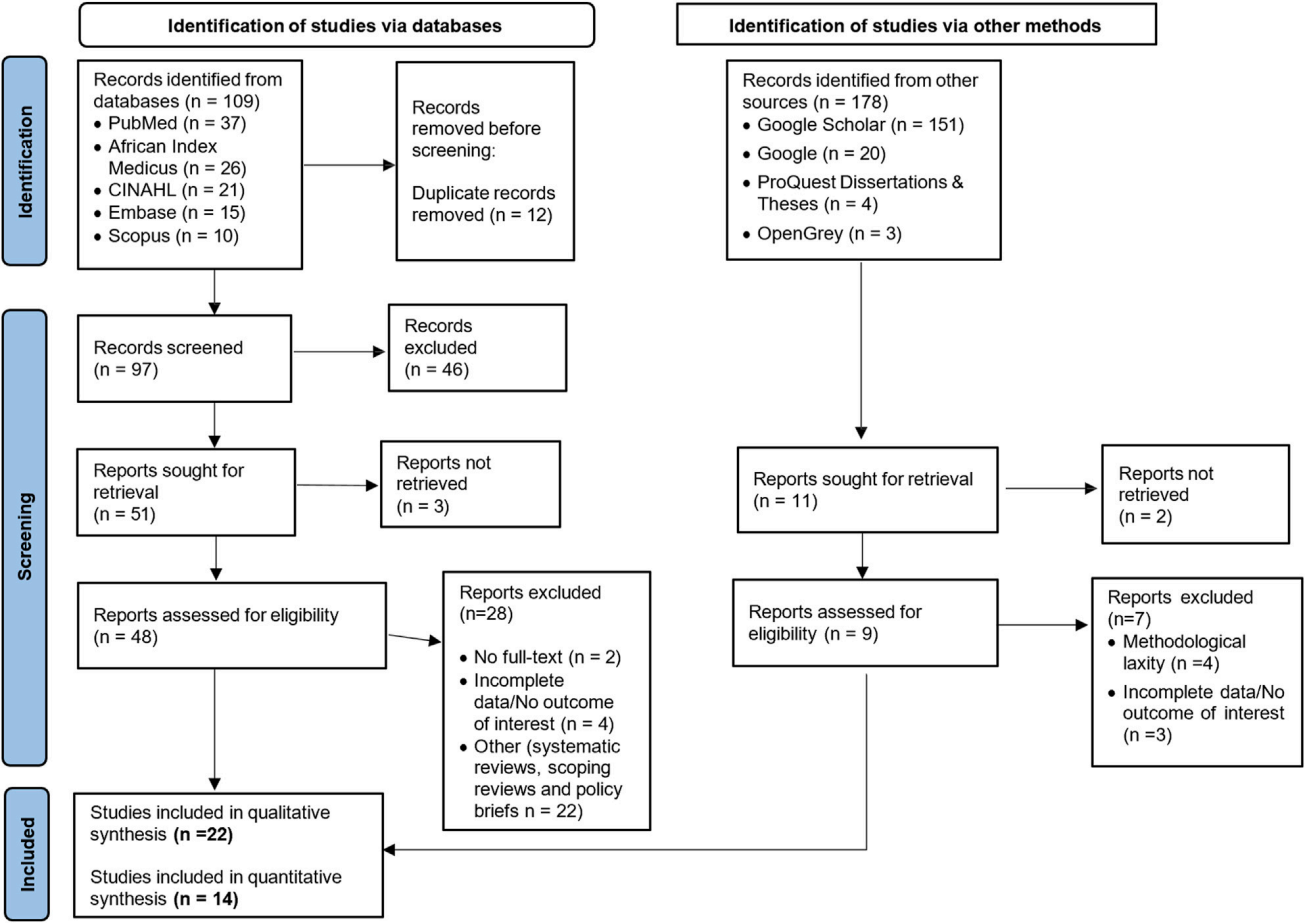
PRISMA diagram showing study selection.

A total of 109 studies were retrieved from various search engines and databases (Figure 1). Of these, 97 remained after removing duplicates. Similarly, about 178 studies were obtained from other sources. After assessing relevance, only 11 studies were retained. Then, 57 studies were evaluated for eligibility. Ultimately, 22 studies were included in the narrative synthesis because they met the criteria of reporting either prevalence data, factors, or indications for the use of HMs. Only 14 studies were included in the meta-analysis, as they provided prevalence data for HMs use consistent with the eligibility criteria, and 8 studies were not included in the final meta-analysis since they did not report the prevalence data (Figure 1).

Out of twenty-two studies that were used in the qualitative synthesis.

### 3.2 Study characteristics

Twenty-two studies included in the analysis involved a total of 5,248 women across 16 administrative regions of Tanzania. Five regions, Mwanza, Mbeya, Kagera, Manyara, Dar es Salaam, and Arusha, had more than one study, while the remaining regions had only one study. The participants’ ages ranged from 15 to 60 years old. About 41% (n = 9) of studies had a low risk of bias, 36% (n = 8) had a moderate risk, and 23% (n = 5) had a high bias risk. The majority, 59%, of the studies were conducted between 2017 and 2025. Most studies (96%) used a cross-sectional design, with interviews used as the common data collection method in 71% of the studies (Table 1).

**TABLE 1:** Characteristics of included studies.

Study ID	Sample size	Data collection method	Mean age (SD)/Range (years)	Study location	Prevalence (%)	Indication for use	Maternal period
August et al. (2015)	132	FGD	19–60	Pwani	N/R	Prevent prolonged labor/induce labor	Pr and Dl
Bakar et al. (2019)	959	IDI	29 (±6)	Unguja	7.5	Easing labor pain	Dl
Bakari and Mahiti (2022)	32	IDI and FGD	18–49	Shinyanga	N/R	Prevent Miscarriage	Pr
Dika et al. (2017)	178	IDI	26.6 (±5.4)	Mwanza	23	Reduction of labor duration, increase milk secretion	Dl
Elewonibi et al. (2021)	109	IDI	16–44	Arusha	33	Unsafe abortion	Pr
Felisian et al. (2022)	12	IDI	15–49	Manyara	N/R	Induce and shorten labor	Dl
Fukunaga et al. (2020)	353	Secondary data analysis	25–49	Kigoma	42.9	Prevent miscarriage, induce and sustain labor, treat stomach pain, and improve fetal health	Pr and Dl
Godlove (2011)	400	IDI	20–40	Mbeya	55	Induce labor	Pr and Dl
Jacob (2010)	253	IDI	15–45	Mbeya	51.3	Easing delivery	Pr and Dl
Katabalo et al. (2022)	381	IDI	18–39	Mwanza	38.3	Prevent miscarriage	Pr
Kessy and Msalale (2020b)	340	IDI	16–36	Tabora	60	Shortening the duration of labor and alleviating labor pain, enhancing milk secretion	Pr and Dl
Mahiti et al. (2015)	105	FGD	14–45	Dodoma	N/R	Assisting/easing deliveries	Pr
Marwa et al. (2018)	372	IDI	18–38	Mwanza	25.3	Treatment of hypertensive disorder of pregnancy	Po
Mbwele et al. (2019)	24	IDI, FGD and Obs	N/R	Manyara	85.7	Induce labor	Dl
Millinga et al. (2022)	372	IDI	27.4 (±5.3)	Morogoro	53.8	Improve low breast milk	Pr
Mwakawanga et al. (2022)	21	FGD	43.3 (9.9)	Dar es Salaam	N/R	Induce and shorten labor/smooth delivery	Pr and Dl
Rasch and Kipingili (2009)	751	IDI	21–30	Dar es Salaam and Kagera	48	Unsafe abortion	Pr
Rasch et al. (2014)	125	IDI and FGD	N/R	Kagera	43	Unsafe abortion	Pr
Siajabu (2009)	200	IDI, Obs and secondary data	20 - >20	Iringa	98	Facilitating labor	Pr
Saruni et al. (2020)	51	IDI and FGDs	18–47	Arusha	N/R	N/R	Pr
Young and Ali (2005)	62	IDI, FGD and Obs	17–60	Pemba	N/R	Induce and shorten labor/smooth delivery	Pr
Sichalwe et al. (2025)	16	IDI	18–39	Mara	N/R	Induce labor	Pr and Po

N/R: not reported; IDI: In-depth interview; FGD: focus group discussion; Obs: Observational; Pr: Pregnancy; Dl: During labor; Po: Postnatal.

Abortion: mean intentional pregnancy termination by skilled provider under hygienic circumstances. Unsafe abortion: mean intentional pregnancy termination by unskilled provider usually under unhygienic circumstances.

### 3.3 Pooled prevalence of HMs use for maternal conditions

**FIGURE 2:**
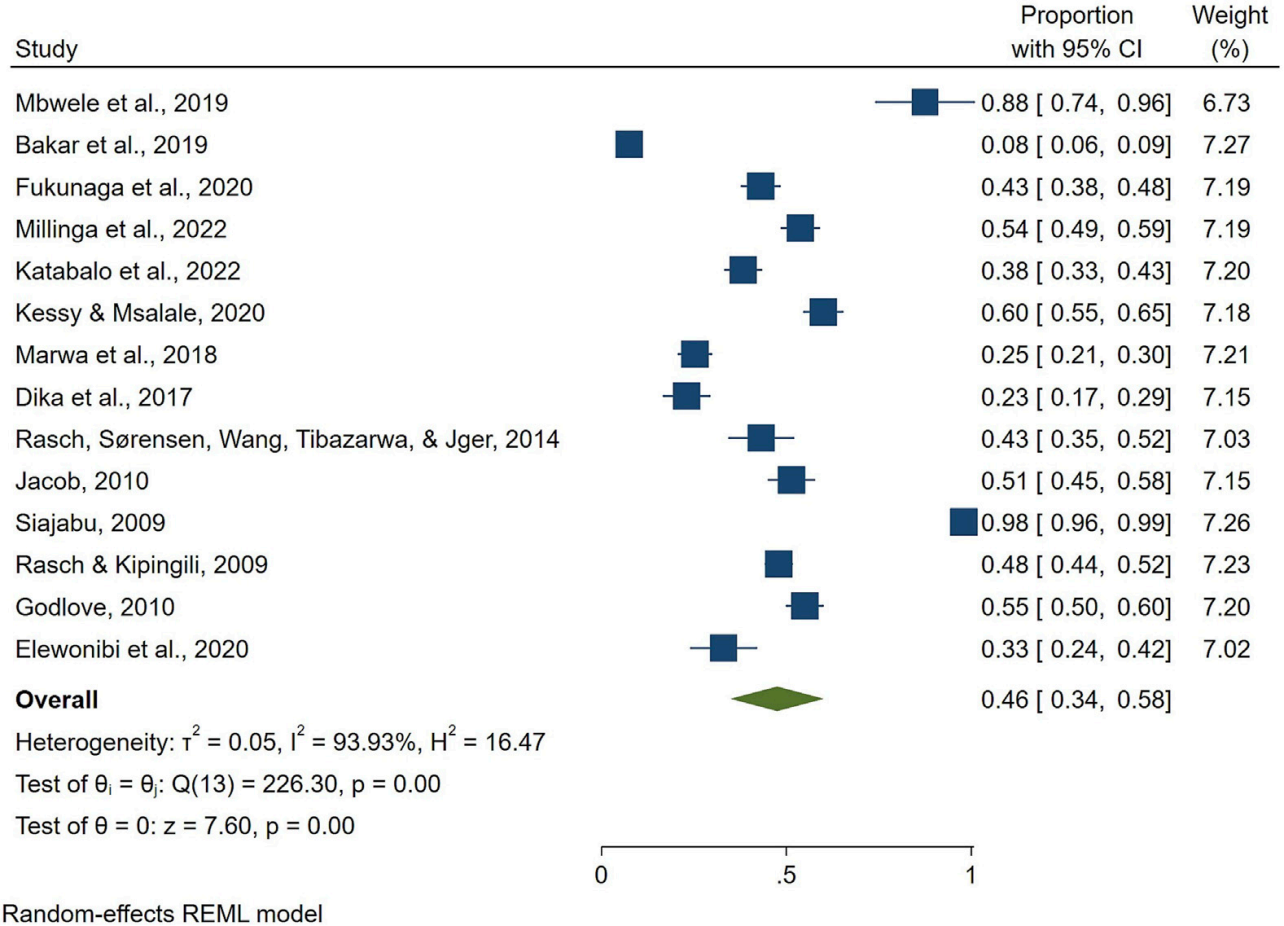
Overall pooled prevalence estimate of HMs use for maternal conditions.

**FIGURE 3:**
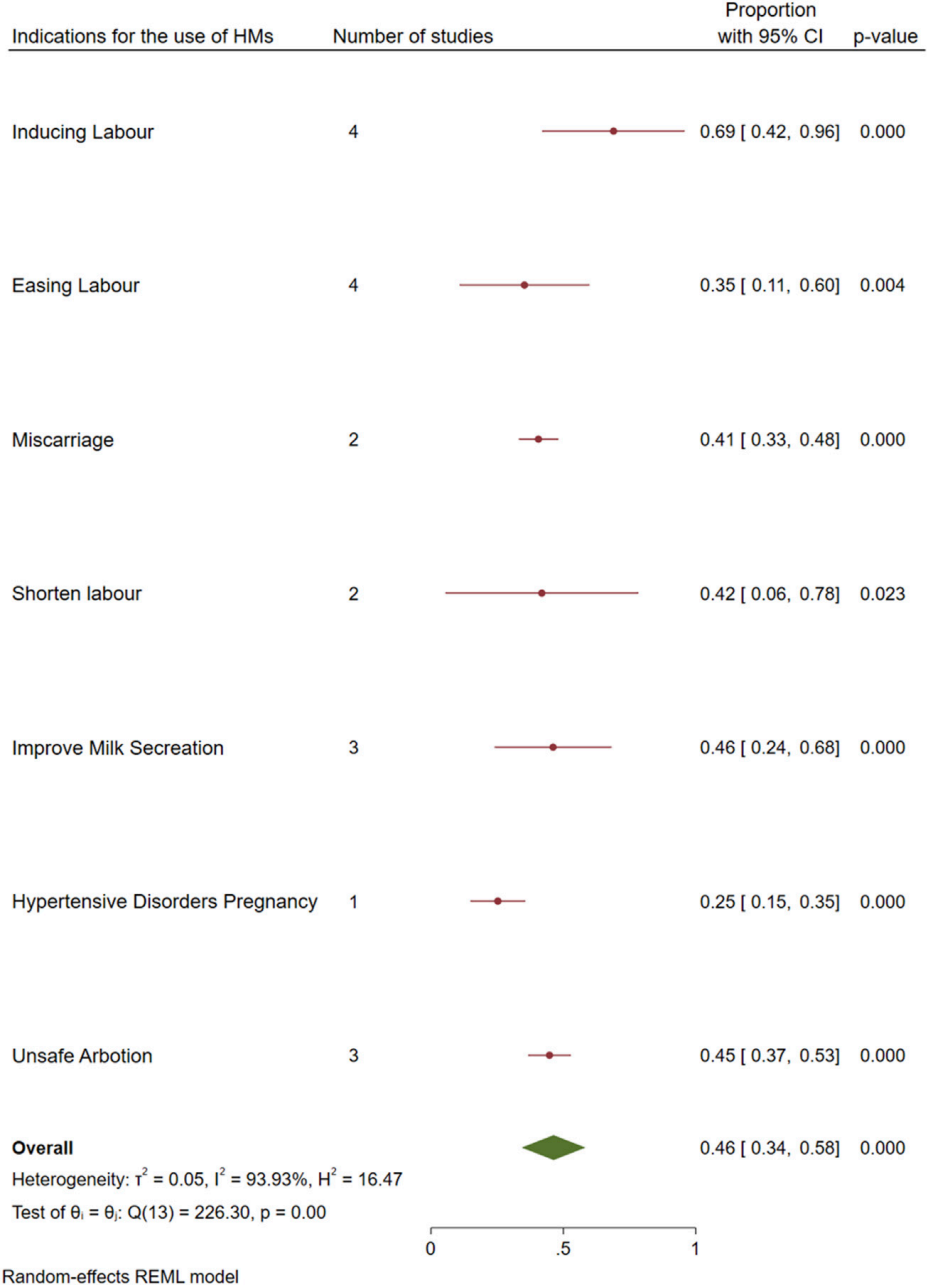
Pooled prevalence of HMs use according to indications.

The overall prevalence of HMs use for maternal conditions in Tanzania was 46% [95%, CI: 34–58]. The *I* ^*2*^ statistic was 93.93%, Q = 226.3, df = 13, P < 0.001, T^2^: 0.05), indicating considerable heterogeneity among the studies (Figure 2). The condition that was managed the most using HMs was labor induction, 69% [95%, CI: 42–96], followed by improving milk secretion 46% [95%, CI: 24–68] and unsafe abortion 45% [95%, CI: 37–53]. The least reported indication for use of HMs was hypertensive disorders during pregnancy, with a proportion of 25% [95%, CI: 15–35] (Figure 3).

**FIGURE 4:**
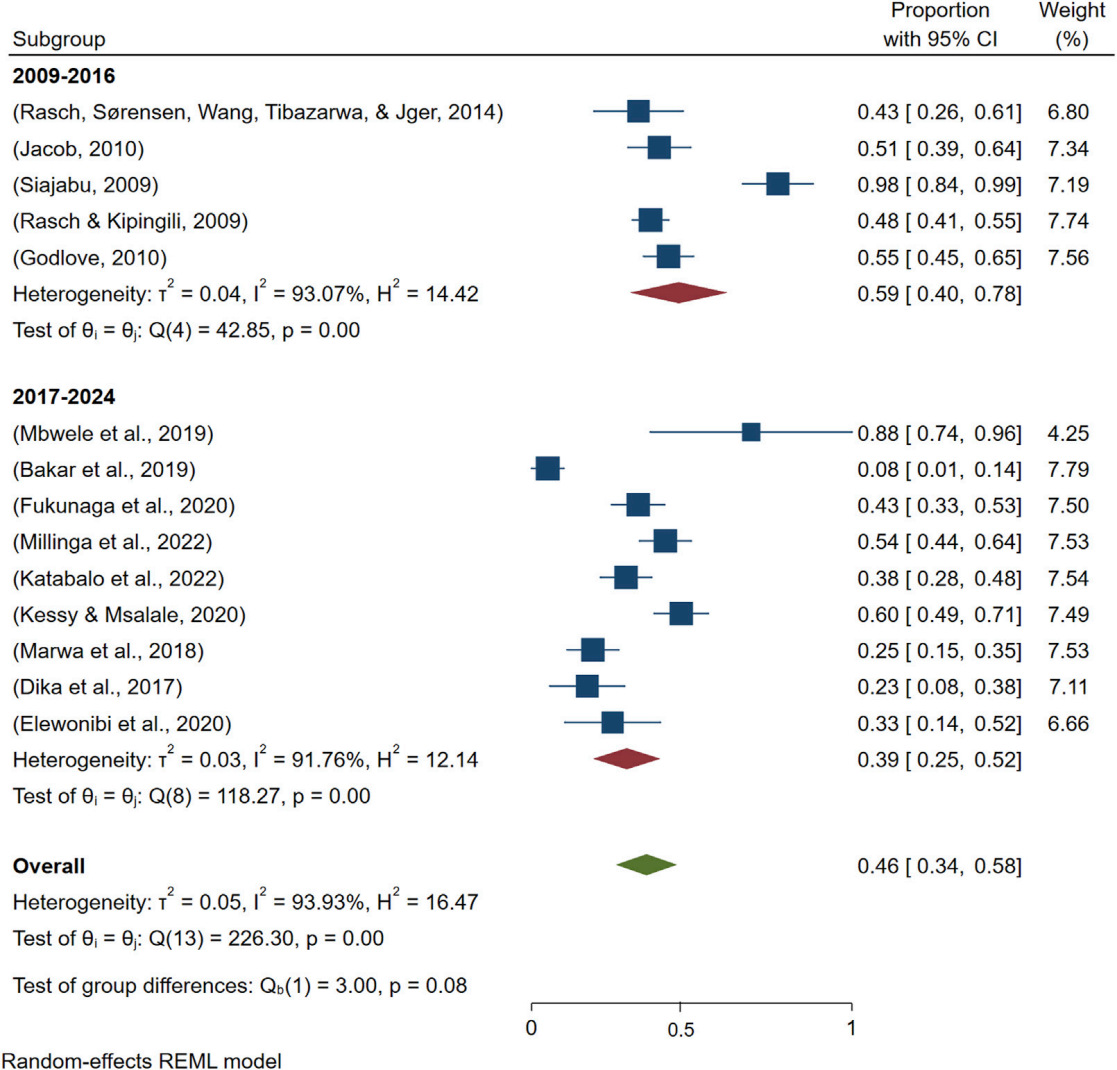
Time-based subgroup meta-analysis on the use of HMs for managing maternal conditions in Tanzania.

Considering the interval of 7 years, the prevalence of HMs use decreased from 59% [95%, CI: 40–78] in (2009–2016) to 39% [95%, CI: 25–52] in (2017–2024) (Figure 4). Furthermore, the p-value of 0.08 suggests that the difference between the two subgroups is not statistically significant at the 0.05 level, although it is close. This implies that while the proportion appears lower in recent studies, the difference may be due to chance, and we cannot confidently conclude a time-based change.

### 3.4 Profile of identified maternal conditions

**FIGURE 5:**
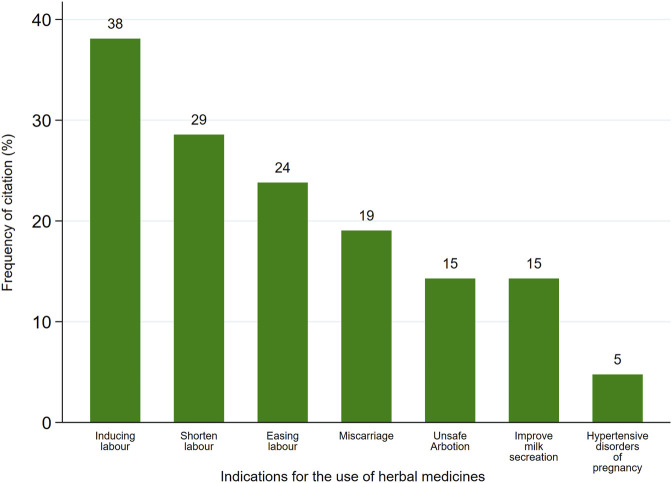
Frequency of citation (%) of maternal conditions managed by HMs.

The review revealed that various HMs managed about seven maternal conditions (Table 1). Furthermore, labor induction was the most frequently cited (38%), followed by Shortening of labor duration (29%), while treating hypertensive disorder of pregnancy was the least frequently cited (5%) (Figure 5).

**FIGURE 6:**
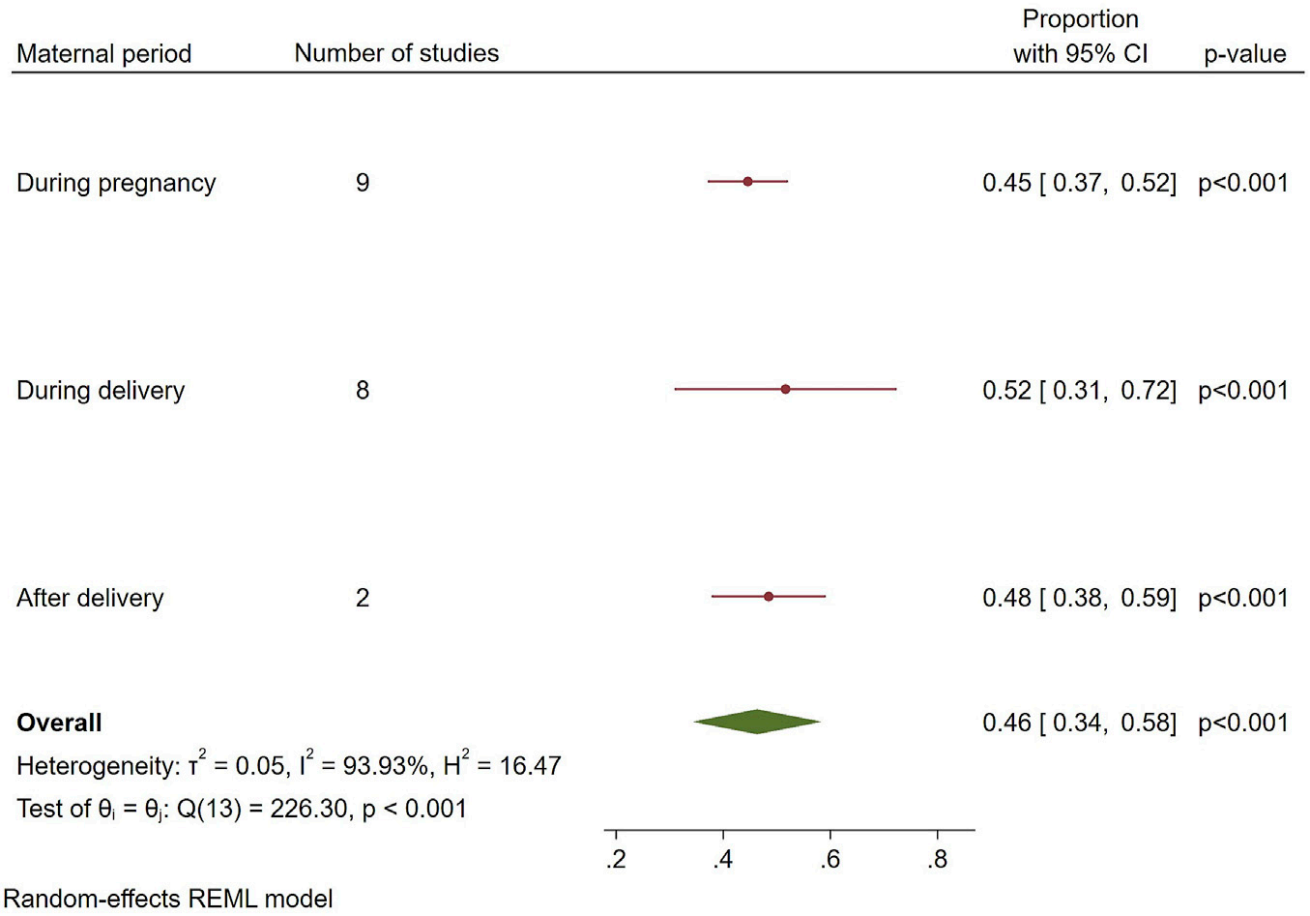
Subgroup meta-analysis on the use of HMs by periods of maternal conditions.

Regarding maternal periods, more than half (52%) of women use HMs during the delivery/labor period, followed by the period after delivery (48%) (Figure 6).

**FIGURE 7:**
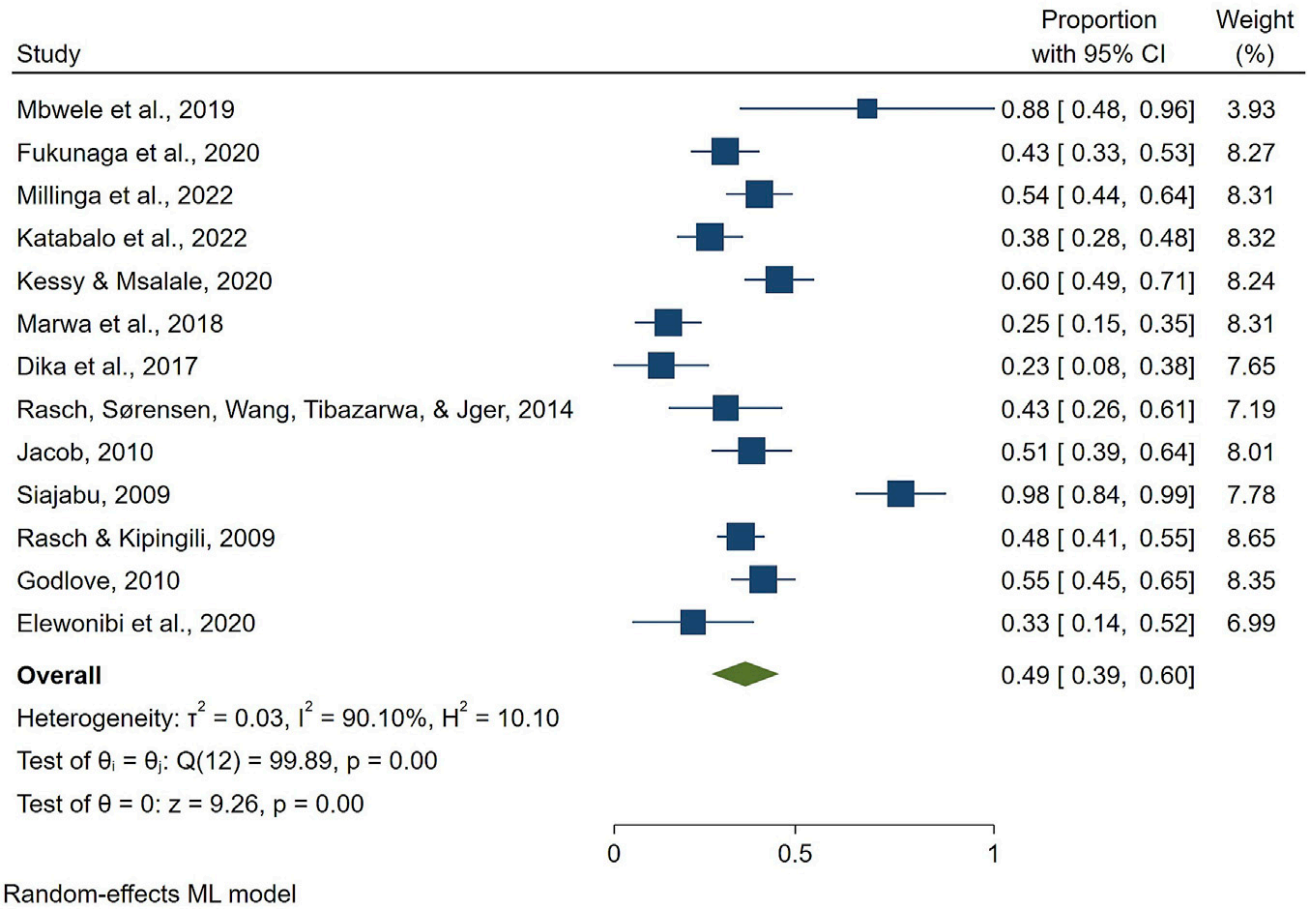
Sensitivity analysis.

Due to the high heterogeneity of the results, we performed a sensitivity analysis. The exclusion of the study by Bakar et al. (2019) reduced the heterogeneity (*I* ^*2*^ = 90.10%, Q = 99.89, df = 12, P < 0.001, T^2^: 0.03), resulting in a slight increase in the overall prevalence for HMs use to 49% [95% CI: 38–60] (Figure 7).

**FIGURE 8:**
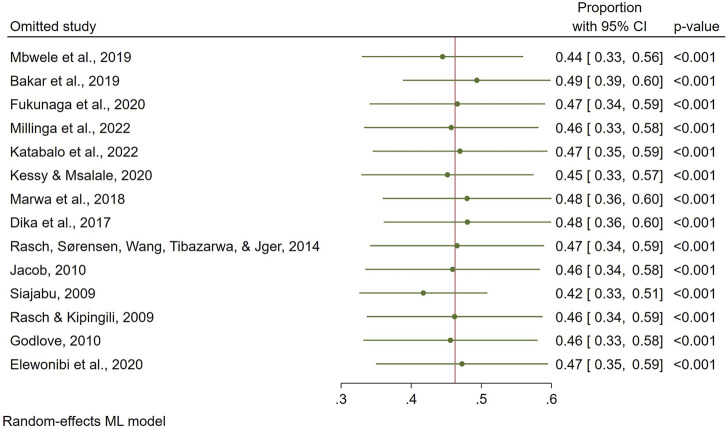
Leave-one-out forest plot.

**FIGURE 9:**
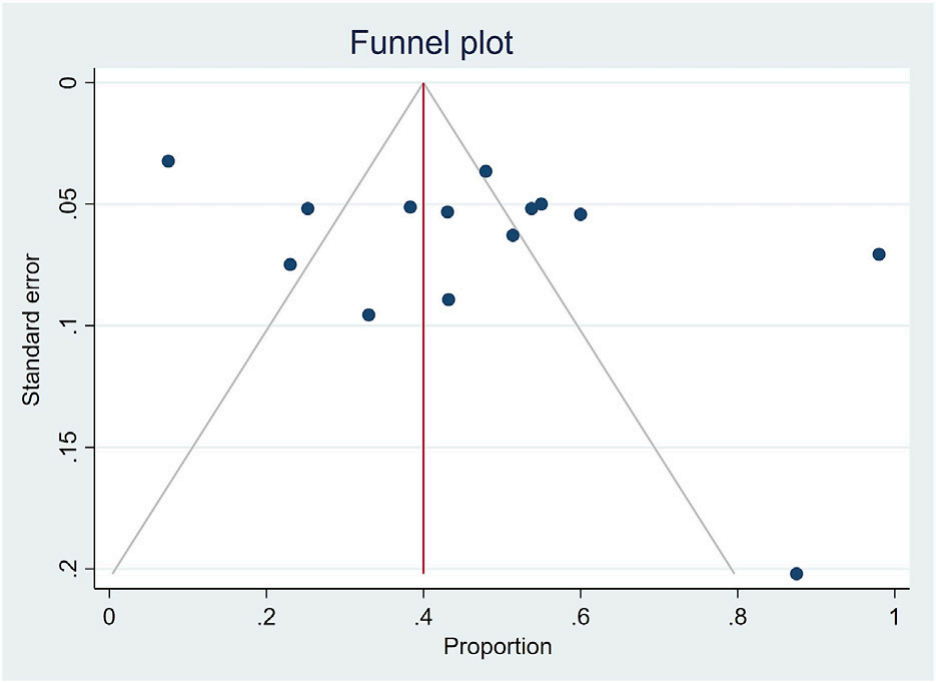
Funnel plot of the results of the prevalence of medicinal herbs use for maternal conditions.

The influence of individual study on the overall prevalence estimate is displayed on a leave-one-out forest plot using the leave-one-out command (Figure 8). On inspection, the funnel plot was symmetrical, as confirmed by Egger’s regression test (P = 0.119), indicating the absence of small-study effects (publication bias) (Figure 9).

### 3.5 Meta-regression analyses

**FIGURE 10:**
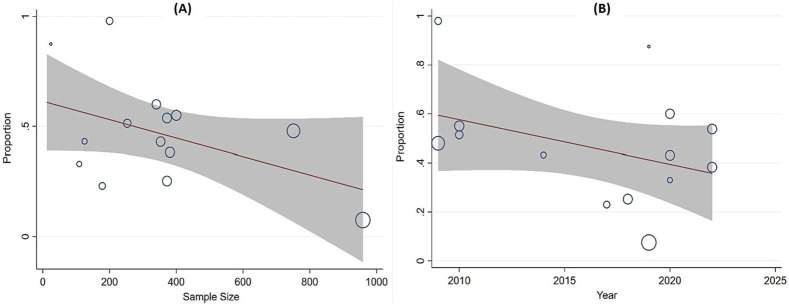
Meta-regression analysis of the prevalence of HMs use against **(A)** sample size and **(B)** year of publication, respectively.

We applied meta-regression to assess the potential effect of factors on the heterogeneity of the prevalence of HMs use for maternal conditions. The investigated factors include sample size and year of publication. The finding indicated that the sample size and year of publication have no significant influence on the prevalence of HMs use for maternal conditions (P = 0.076) and (P = 0.128), respectively (Figure 10).

### 3.6 Patterns of medicinal herbs use for maternal conditions

**FIGURE 11:**
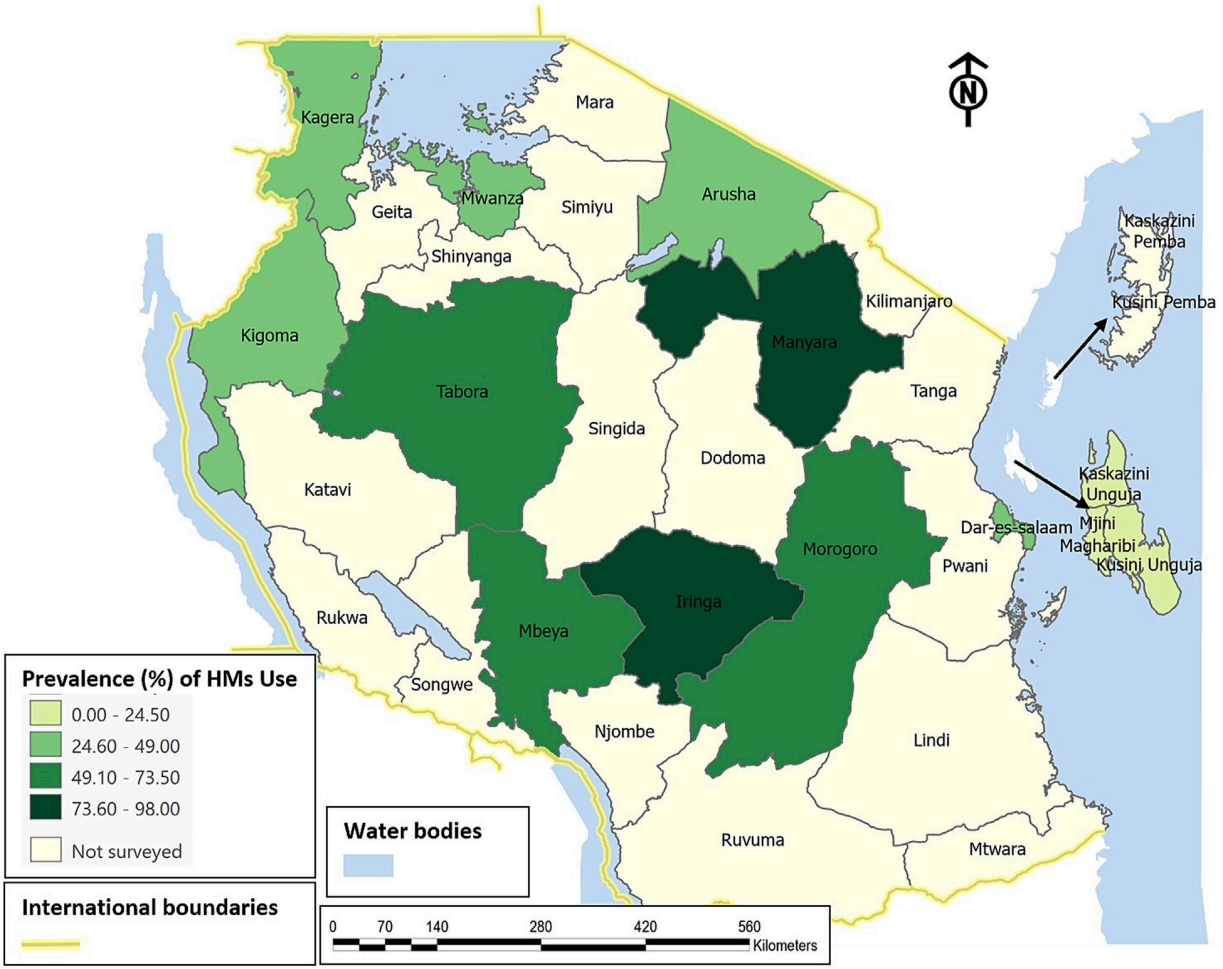
Spatial distribution on prevalence of HMs use for maternal conditions across regions of Tanzania.

A choropleth map in (Figure 11) shows the spatial patterns of the prevalence of HMs use for managing maternal conditions across regions in Tanzania. A high level of heterogeneity is observed, with Iringa and Manyara regions presenting the highest prevalence (>73.5%), Tabora, Mbeya and Morogoro regions ranging between (49.1%–73.5%), and the lowest (below 24.5%) prevalence observed in Unguja.

### 3.7 Factors influencing HMs use for maternal conditions

Narrative synthesis revealed that cultural and traditional customs, strong ties to the local knowledge, low level of education and illiteracy, distance from health facilities, influence from older family members (i.e., elders), place of residence (i.e., rural), increasing age, parity (i.e., increasing number of children), low economic status, presence of traditional birth attendants (TBA’s) and negative beliefs on modern drugs are important factors associated with HMs use for maternal conditions (Table 2).

**TABLE 2:** Socio-demographic factors influencing HMs use in maternal conditions.

Factors	Narratives	Citations
Cultural, customs, traditional beliefs and Strong ties to the local knowledge	Women are strongly holding to the traditional believes and local knowledge concerning to the use of HMs for managing maternal conditions, also affirmed that HMs are better than hospital drugs	August et al. (2015), Elewonibi et al. (2021), Felisian et al. (2022), Mbwele et al. (2019), Mwakawanga et al. (2022), Siajabu (2009), Sichalwe et al. (2025), Young and Ali (2005)
Level of education	Illiteracy and low level of education is associated with the use of HMs for managing maternal conditions	Bakar et al. (2019), Katabalo et al. (2022), Mahiti et al. (2015), Marwa et al. (2018), Millinga et al. (2022), Rasch and Kipingili (2009)
Religion	Majority of participants who are exposed to the use HMs were Muslims	Bakar et al. (2019)
Age	The use of herbal drugs was significantly associated with the age of respondents, as the age increases tendency of using herbal drugs increased	Fukunaga et al. (2020), Godlove (2011), Katabalo et al. (2022)
Economic status and place of delivery	The use herbal drugs were significantly associated with the low-economic status and place of delivery (i.e., home delivery)	Fukunaga et al. (2020)
Parity or number of children	It is noted that HMs use was significantly associated with the number of children. Women who they have high parity were largely associated with the use of HMs	Godlove (2011)
Distance or accessibility of health facilities	It is noted that the use of HMs was associated with the distance to nearest health facility	Felisian et al. (2022), Fukunaga et al. (2020)
Residence	People from rural areas commonly exposed to the use of HMs to manage maternal conditions compared to those from urban areas	Katabalo et al. (2022)
Occupation	It is observed that women who are exposed to the use of HMs were from rural areas and engage farming activities	Katabalo et al. (2022), Mahiti et al. (2015)
Influence from older people	The influence from older family/community members (e.g., elders and mothers) seems to be the common factor for the use of HMs	Dika et al. (2017), Godlove (2011), Sichalwe et al. (2025)
Services provided by TBA’s and perceived safety of herbs	Presence of traditional birth attendants and belief that HMs are safer and natural compared to modern drugs makes women to rely on HMs	Saruni et al. (2020), Sichalwe et al. (2025)
Marital status	Being married were largely associated with the use of HMs	Dika et al. (2017)
Modern medicine and health facilities are not enough to accommodate the practice	Women believed that modern medicine alone could not address all of their pregnancy-related health issues	Felisian et al. (2022), Godlove (2011), Sichalwe et al. (2025)

## 4 Discussion

To the best of our knowledge, this systematic review and meta-analysis is the first to provide qualitative and quantitative synthesis estimates of the national prevalence of and factors influencing HMs use for maternal conditions in Tanzania. The overall national prevalence of HMs use for maternal conditions is 46%. This means that, for every five Tanzanian women, at least two are using HMs for various maternal conditions. This information can assist in the planning of the management of maternal conditions by ensuring that mechanisms are in place to screen women for previous exposure to HMs. Such practice could enhance healthcare professionals’ understanding of the profile of HMs used by women while planning for conventional interventions critical to preventing potential adverse drug-herbal interactions.

While this is the first systematic review in the East African region, our findings are congruent with those reported in Ethiopia, where about 47.77% of women use HMs (Adane et al., 2020). The similarity observed between the two countries could indicate the shared socio-cultural beliefs and practices as these countries are located proximal to each other in the Eastern and north-eastern African regions. Regarding the maternal periods, contrary to our findings that the pooled prevalence of HMs use during pregnancy is 45%, a systematic review and meta-analysis of the prevalence of HMs use during pregnancy globally revealed an average of 32.4% (Heydarpour et al., 2022). The differences observed between the two systematic reviews could reflect the diverse socio-cultural context of the study population. While our systematic review focused on national prevalence from data on the same country, the latter pooled prevalence across countries with diverse cultural backgrounds. Nonetheless, these findings corroborate previous findings that women are increasingly using HMs during pregnancy (El Hajj and Holst, 2020; John and Shantakumari, 2015). In recognition of the high demand for HMs among Tanzanian communities and the potential contribution of HMs to accelerate the realization of universal health coverage, in 2022, the Government of Tanzania, through its ministry responsible for health, launched a pilot integration of traditional medicine in a formal healthcare system where HMs for selected diseases are dispensed at formal health facilities (MoH, 2022). This integration has been implemented for more than a year with satisfactory performance, but it has not provided guidelines for HMs to manage maternal conditions. Hence, the findings of this systematic review and meta-analysis could inform integration efforts on the possibility of expanding its scope to include maternal conditions frequently managed by HMs.

While several communities regard HMs as natural and may not harm pregnant women, recent studies have indicated some adverse effects associated with the use of certain HMs during pregnancy (Sarecka-hujar, 2022). Thus, the higher prevalence of HMs used during pregnancy in our study calls for deliberate efforts to identify and understand their safety and efficacy in humans.

The grounds for the frequent usage of HMs could be attributed to culture and beliefs, poverty, long distance to health facilities, high cost of conventional medicines, easy accessibility, and acceptability of HMs (Mudonhi and Nunu, 2022; Peprah et al., 2019). Again, the popularity of HMs use among women in maternal conditions could be mainly attributed to the belief that herbal products are natural and safe, with fewer adverse events compared to conventional drugs (Barnes et al., 2018; Ernst, 2002). In the current study, cultural and traditional customs, strong ties to local knowledge, low level of education and illiteracy, influence from older family members (e.g., elders), distance from health facilities, place of residence (e.g., rural), increasing age, low economic status, presence of traditional birth attendants (TBAs), and perceived safety of herbs are the driving factors for the usage of HMs among women.

There is an unequal distribution of HMs used among women in maternal conditions across different regions in Tanzania. Furthermore, when compared to other regions in Tanzania, Iringa and Manyara had the highest prevalence of HMs use for maternal conditions, accounting for more than 73.5 percent. This implies that efforts to create awareness among healthcare professionals of the substantial use of HMs by women for various maternal conditions have become the need of the hour. Interestingly, Zanzibar had the lowest prevalence, accounting for eight percent; this does not necessarily mean that the prevalence is lower in that archipelago; it might be because of the dearth of studies investigating HMs use for maternal conditions as only one study contributed to the overall pooled prevalence.

Contrary to the belief that the general population in Tanzania is increasingly using HMs for healthcare needs, our findings revealed a decreasing trend in the use of HMs for maternal conditions. However, the observed trend was not statistically significant and could likely be explained by the variation in a number of studies over time and settings or the growing inaccessibility of HMs in recent years. Nevertheless, such a decreasing trend raised critical research questions that call for researchers to assess the national prevalence of HMs use among women for maternal conditions using an appropriate sample size to validate the observed trend and also to determine barriers and facilitators of use.

## 5 Limitations of the study

Our systematic review and meta-analysis included data from sixteen out of thirty-one administrative regions in Tanzania. This may have led to insufficient representation from other regions, potentially underestimating the current national estimate. The missing regions likely represent populations with different practices regarding HMs use. However, the current national estimate offers a baseline that can serve in the absence of reliable national prevalence data for planning and managing maternal conditions. Additionally, the meta-analysis used survey data that could have been affected by biases such as social desirability and self-reporting, which might have influenced the national estimate. Nevertheless, efforts were made to assess bias risk, and only studies designed to minimize bias were included. Some relevant studies were excluded because the authors did not respond to follow-up emails despite three reminders over 2 months, which may have slightly limited our review scope. The lack of data from other regions highlights the need for further research on documenting HMs use among local communities to achieve better representation. Due to the limited availability of high-quality studies, some included studies had a significant risk of bias. This could affect the reliability of the overall findings, so the results should be interpreted carefully. However, their inclusion was deemed necessary to ensure comprehensive coverage of existing evidence. Furthermore, the narrative synthesis method used in identifying factors influencing the use of HMs among women did not support causal inference. The synthesis captured perceived or reported associations rather than statistically validated relationships. The results should therefore be interpreted as indicative of commonly reported influences within the available literature, not as definitive or causal effects.

## 6 Conclusion

Based on our findings, about 46% of women in Tanzania use some form of HMs to manage maternal conditions, and more women use them during delivery/labor and after delivery. Furthermore, the most commonly and frequently cited maternal condition managed by HMs is labor induction. Policymakers and healthcare professionals could use the national estimate of HMs use for planning the management of maternal conditions in Tanzania. However, data from better-designed ethnomedical surveys from all regions are still needed for a robust, generalizable estimate.

## Data Availability

The original contributions presented in the study are included in the article/Supplementary Material, further inquiries can be directed to the corresponding author.

## Abbreviations

CI, Confidence interval; HMs, Herbal Medicines; NIMR, National Institute for Medical Research; TBA’s, Traditional Birth Attendants.

## References

[B1] Adane F. Seyoum G. Alamneh Y. M. Abie W. Desta M. Sisay B. (2020). Herbal medicine use and predictors among pregnant women attending antenatal care in Ethiopia: a systematic review and meta-analysis. BMC Pregnancy Childbirth 20, 157. 10.1186/s12884-020-2856-8 32164603 PMC7069203

[B2] Attah A. F. O’Brien M. Koehbach J. Sonibare M. A. Moody J. O. Smith T. J. (2012). Uterine contractility of plants used to facilitate childbirth in Nigerian ethnomedicine. J. Ethnopharmacol. 143, 377–382. 10.1016/j.jep.2012.06.042 22766472 PMC3430860

[B3] August F. Pembe A. B. Kayombo E. Mbekenga C. Axemo P. Darj E. (2015). Birth preparedness and complication readiness - a qualitative study among community members in rural Tanzania. Glob. Health Action 8, 26922. 10.3402/gha.v8.26922 26077145 PMC4468055

[B4] Bakar R. R. Manongi R. N. Mmbaga B. T. Nielsen B. B. (2019). Perinatal mortality and associated risk factors among singleton babies in Unguja Island, zanzibar. Zanzibar. Health Irvine. Calif. 11, 91–107. 10.4236/health.2019.111010

[B5] Bakari H. Mahiti G. R. (2022). Factors for late initiation of antenatal care in Kahama Municipal, Tanzania. Eur. J. Clin. Med. 3, 1–10. 10.24018/clinicmed.2022.3.1.149

[B6] Barnes L. A. J. Barclay L. McCaffery K. Aslani P. (2018). Complementary medicine products used in pregnancy and lactation and an examination of the information sources accessed pertaining to maternal health literacy: a systematic review of qualitative studies. BMC Complement. Altern. Med. 18, 229. 10.1186/s12906-018-2283-9 30064415 PMC6069845

[B7] Begg C. B. Mazumdar M. (1994). Operating characteristics of a rank correlation test for publication bias. Biometrics 50, 1088–1101. 10.2307/2533446 7786990

[B8] Borenstein M. Hedges L. V. Higgins J. P. T. Rothstein H. R. (2010). A basic introduction to fixed-effect and random-effects models for meta-analysis. Res. Synth. Methods 1, 97–111. 10.1002/jrsm.12 26061376

[B9] Deeks J. J. Higgins J. P. Altman D. G. (2008). “Analysing data andundertaking meta-analyses,” in Cochrane handbook for systematic reviews of interventions: cochrane book series. Editors Deeks J. J. Higgins J. P. Altman D. G. (Chichester (UK): John Wiley & Sons), 244–649. 10.1002/9780470712184

[B10] Dika H. I. Dismas M. Iddi S. Rumanyika R. (2017). Prevalent use of herbs for reduction of labour duration in Mwanza, Tanzania: are obstetricians aware? Tanzan. J. Health Res. 19, 1–8. 10.4314/thrb.v19i2.5

[B11] Egger M. Davey Smith G. Schneider M. Minder C. (1997). Bias in meta-analysis detected by a simple, graphical test. BMJ 315, 629–634. 10.1136/BMJ.315.7109.629 9310563 PMC2127453

[B12] El Hajj M. Holst L. (2020). Herbal medicine use during pregnancy: a review of the literature with a special focus on sub-Saharan Africa. Front. Pharmacol. 11, 866. 10.3389/fphar.2020.00866 32581815 PMC7296102

[B13] Elewonibi B. Amour C. Gleason S. Msuya S. Canning D. Shah I. (2021). Estimating the lifetime incidence of induced abortion and understanding abortion practices in a Northeastern Tanzania community through a household survey. Contraception 103, 127–131. 10.1016/j.contraception.2020.10.013 33098850

[B14] Ernst E. (2002). Herbal medicinal products during pregnancy: are they safe? BJOG 109, 227–235. 10.1111/j.1471-0528.2002.t01-1-01009.x 11950176

[B15] Felisian S. Mushy S. E. Tarimo E. A. M. Kibusi S. M. (2022). Sociocultural practices and beliefs during pregnancy, childbirth, and postpartum among indigenous pastoralist women of reproductive age in Manyara, Tanzania: a descriptive qualitative study. BMC Womens Health 23, 123. 10.1186/s12905-023-02277-4 36959588 PMC10035110

[B16] Fukunaga R. Morof D. Blanton C. Ruiz A. Maro G. Serbanescu F. (2020). Factors associated with local herb use during pregnancy and labor among women in Kigoma region, Tanzania, 2014-2016. BMC Pregnancy Childbirth 20, 122–11. 10.1186/s12884-020-2735-3 32085731 PMC7035699

[B17] Godlove M. J. (2011). “Prevalence of herbal medicine use and associated factors among boer, pregnant women attending antenatal clinic at Mbeya refferal hospital in 2010,”. M. Sc. Thesis (Dar es salaam, Tanzania: Muhimbili Univ. Heal. Allied Sci.).

[B18] Graham W. J. Cairns J. Bhattacharya S. Bullough C. H. W. Quayyum Z. Rogo K. (2006). “Maternal and perinatal conditions,” in Disease control priorities in developing countries, 499–530.

[B19] Heydarpour F. Heydarpour S. Dehghan F. Mohammadi M. Farzaei M. H. (2022). Prevalence of medicinal herbs use during pregnancy in the world: a systematic review and meta-analysis. J. Chem. Heal. Risks 12, 183–196. 10.22034/jchr.2020.1890228.1084

[B20] Illamola S. M. Amaeze O. U. Krepkova L. V. Birnbaum A. K. Karanam A. Job K. M. (2020). Use of herbal medicine by pregnant women: what physicians need to know. Front. Pharmacol. 10, 1483. 10.3389/fphar.2019.01483 31998122 PMC6962104

[B21] Jacob F. (2010). Factors associated with the use of herbal drugs during pregnancy in rungwe district, Mbeya Region, Tanzania.

[B22] John L. J. Shantakumari N. (2015). Herbal medicines use during pregnancy: a review from the Middle East. Oman Med. J. 30, 229–236. 10.5001/omj.2015.48 26366255 PMC4561638

[B23] Katabalo D. M. Robert D. N. Mwita S. Minja W. V. Abbas S. (2022). Self-medication during first trimester among pregnant women attending antenatal care clinic at a district hospital in Mwanza, north-western Tanzania. J. Preg Child. Heal 5, 119. 10.29011/JPCH-119.100019

[B24] Kessy A. T. Msalale G. C. (2020a). Understanding forgotten exposures towards achieving sustainable development goal 3: the case of herbal medicine use in Tanzania. 10.1186/s12884-021-03741-5 PMC8017693 33794794

[B25] Kessy A. T. Msalale G. C. (2020b). Prevalence and predictors of use of herbal medicines among the Most recently delivered mothers in Tabora municipality, Tanzania.

[B26] Mahiti G. R. Mkoka D. A. Kiwara A. D. Mbekenga C. K. Hurtig A.-K. Goicolea I. (2015). Women’s perceptions of antenatal, delivery, and postpartum services in rural Tanzania. Glob. Health Action 8, 28567. 10.3402/gha.v8.28567 26498576 PMC4617868

[B27] Marwa K. J. Njalika A. Ruganuza D. Katabalo D. Kamugisha E. (2018). Self-medication among pregnant women attending antenatal clinic at makongoro health centre in Mwanza, Tanzania: a challenge to health systems. BMC Pregnancy Childbirth 18, 16–18. 10.1186/s12884-017-1642-8 29310609 PMC5759229

[B28] Mbwele B. Mwaitebele U. K. Kahsay A. Kihako O. P. Luhunga S. J. Zuberi M. A. (2019). A situational analysis of home delivery among Maasai communities of Orkesumet, Northern Tanzania: the qualitative evidences. J. Heal. Med. Sci. 2, 1–13. 10.31014/aior.1994.02.01.13

[B29] Millinga V. P. Im H. B. Hwang J. H. Choi S. J. Han D. (2022). Use of herbal medicines among breastfeeding mothers in Tanzania: a cross-sectional study. Front. Pharmacol. 13, 751129. 10.3389/fphar.2022.751129 35571105 PMC9096166

[B30] MoH (2022). Traditional medicine standard treatment guidelines.

[B31] MoH (2023). Tanzania demographic and health survey and malaria indicator survey 2022 key indicators report. Dodoma: National Bureau of Statistics.

[B32] Moher D. Shamseer L. Clarke M. Ghersi D. Liberati A. Petticrew M. (2015). Preferred reporting items for systematic review and meta-analysis protocols (PRISMA-P) 2015 statement. Syst. Rev. 4, 1. 10.1186/2046-4053-4-1 25554246 PMC4320440

[B33] Mudonhi N. Nunu W. N. (2022). Traditional medicine utilisation among pregnant women in sub-saharan African countries: a systematic review of literature. Inq. J. Heal. Care Organ. Provis. Financ. 59, 00469580221088618. 10.1177/00469580221088618 35506677 PMC9073130

[B34] Musarandega R. Nyakura M. Machekano R. Pattinson R. Munjanja S. P. (2021). Causes of maternal mortality in Sub-Saharan Africa: a systematic review of studies published from 2015 to 2020. J. Glob. Health 11, 04048. 10.7189/jogh.11.04048 34737857 PMC8542378

[B35] Mwakawanga D. L. Mwilike B. Kaneko M. Shimpuku Y. (2022). Local knowledge and derived practices of safety during pregnancy, childbirth and postpartum: a qualitative study among nurse-midwives in urban eastern Tanzania. BMJ Open 12, e068216. 10.1136/bmjopen-2022-068216 36521900 PMC9756159

[B36] Oyston C. Baker P. N. (2020). Current challenges in pregnancy-related mortality. Obstet. Gynaecol. Reprod. Med. 30, 55–61. 10.1016/J.OGRM.2019.11.003

[B37] Page M. J. McKenzie J. E. Bossuyt P. M. Boutron I. Hoffmann T. C. Mulrow C. D. (2021). The PRISMA 2020 statement: an updated guideline for reporting systematic reviews. BMJ 372, n71–n79. 10.1136/bmj.n71 33782057 PMC8005924

[B38] Peprah P. Agyemang-Duah W. Arthur-Holmes F. Budu H. I. Abalo E. M. Okwei R. (2019). “We are nothing without herbs”: a story of herbal remedies use during pregnancy in rural Ghana. BMC Complement. Altern. Med. 19, 65–12. 10.1186/s12906-019-2476-x 30876425 PMC6419816

[B39] Prasad N. Mwakatundu N. Dominico S. Masako P. Mongo W. Mwanshemele Y. (2022). Improving maternal and reproductive health in Kigoma, Tanzania: a 13-Year initiative. Glob. Heal. Sci. Pract. 10, e2100484–17. 10.9745/GHSP-D-21-00484 35487559 PMC9053157

[B40] Rasch V. Kipingili R. (2009). Unsafe abortion in urban and rural Tanzania: method, provider and consequences. Trop. Med. Int. Health 14, 1128–1133. 10.1111/j.1365-3156.2009.02327.x 19573141

[B41] Rasch V. Sørensen P. H. Wang A. R. Tibazarwa F. Jäger A. K. (2014). Unsafe abortion in rural Tanzania–the use of traditional medicine from a patient and a provider perspective. BMC Pregnancy Childbirth 14, 1–8. 10.1186/s12884-015-0794-7 25524498 PMC4279892

[B42] Rodgers M. Sowden A. Petticrew M. Arai L. Roberts H. Britten N. (2009). Testing methodological guidance on the conduct of narrative synthesis in systematic reviews: effectiveness of interventions to promote smoke alarm ownership and function. Evaluation 15, 49–73. 10.1177/1356389008097871

[B43] Sarecka-hujar B. Szulc-Musioł B. (2022). Herbal medicines — are they effective and safe during pregnancy. Pharmaceutics 14, 171–27. 10.3390/pharmaceutics14010171 35057067 PMC8802657

[B44] Saruni K. Mosley P. D. Saruni K. Lenga B. (2020). Factors influencing adoption of facility-assisted delivery-a qualitative study of women and other stakeholders in a Maasai community in Ngorongoro District, Tanzania. BMC Pregnancy Childbirth 20, 100–116. 10.1186/s12884-020-2728-2 32050919 PMC7014728

[B45] Shewamene Z. Dune T. Smith C. A. (2017). The use of traditional medicine in maternity care among African women in Africa and the diaspora: a systematic review. BMC Complement. Altern. Med. 17, 382. 10.1186/s12906-017-1886-x 28768534 PMC5541739

[B46] Shija A. E. Msovela J. Mboera L. E. G. (2011). Maternal health in fifty years of Tanzania Independence: challenges and opportunities of reducing maternal mortality. Tanzan. J. Health Res. 13, 352–364. 10.4314/thrb.v13i5.5 26591990

[B47] Siajabu M. J. (2009). Home deliveries: factors influencing them and their impact on maternal and infant mortality in Songea rural district.

[B48] Sichalwe M. M. mhinte S. R. Kimaro R. R. (2025). Motivations for herbal medicine use during pregnancy and childbirth in Butiama District, Tanzania: a phenomenological qualitative study. Heal. Sci. Rep. 8, e70895. 10.1002/hsr2.70895 40510525 PMC12158660

[B49] Tanzania National Bureau of Statistics (2013). Tanzania population and housing census.

[B50] Young S. L. Ali S. M. (2005). Linking traditional treatments of maternal anaemia to iron supplement use: an ethnographic case study from Pemba Island, Zanzibar. Matern. Child. Nutr. 1, 51–58. 10.1111/j.1740-8709.2004.00002.x 16881879 PMC6874392

