# Supplementary material for: Prevalence of herbal medicine use for maternal conditions in Tanzania: a systematic review and meta-analysis
Source: Front Pharmacol. 2025 Sep 2;16:1637891. doi: 10.3389/fphar.2025.1637891 (PMC12436274; doi:10.3389/fphar.2025.1637891)
Supplement: Supplementary file 1 [file Supplementaryfile1.docx]

**Search strategy**

Search: **(Prevalence OR magnitude OR Use OR percent OR trend) AND ("herbal medicine" OR "plant extracts" OR herb* OR "traditional medicine" OR "herbal remedies" OR "medicinal plant") AND ("maternity" OR "maternal conditions" OR mother* OR pregnancy OR pregnant OR perinatal) AND (Tanzania OR tanga OR Arusha OR "Dar es Salaam" OR Dodoma OR Geita OR Iringa OR Kagera OR Katavi OR Kigoma OR Kilimanjaro OR Lindi OR Manyara OR Mara OR Mbeya OR Morogoro OR Mtwara OR Mwanza OR Njombe OR "Pemba North" OR "Pemba South" OR Pwani OR Rukwa OR Ruvuma OR Shinyanga OR Simiyu OR Singida OR Tabora OR Tanga OR "Zanzibar North" OR "Zanzibar South" OR "Zanzibar West")**

("epidemiology"[MeSH Subheading] OR "epidemiology"[All Fields] OR "prevalence"[All Fields] OR "prevalence"[MeSH Terms] OR "prevalance"[All Fields] OR "prevalences"[All Fields] OR "prevalence s"[All Fields] OR "prevalent"[All Fields] OR "prevalently"[All Fields] OR "prevalents"[All Fields] OR ("magnitude"[All Fields] OR "magnitudes"[All Fields]) OR ("statistics and numerical data"[MeSH Subheading] OR ("statistics"[All Fields] AND "numerical"[All Fields] AND "data"[All Fields]) OR "statistics and numerical data"[All Fields] OR "use"[All Fields]) OR ("percent"[All Fields] OR "percents"[All Fields]) OR ("trend"[All Fields] OR "trended"[All Fields] OR "trending"[All Fields] OR "trends"[MeSH Subheading] OR "trends"[All Fields])) AND ("herbal medicine"[All Fields] OR "plant extracts"[All Fields] OR "herb*"[All Fields] OR "traditional medicine"[All Fields] OR "herbal remedies"[All Fields] OR "medicinal plant"[All Fields]) AND ("maternity"[All Fields] OR "maternal conditions"[All Fields] OR "mother*"[All Fields] OR ("pregnancy"[MeSH Terms] OR "pregnancy"[All Fields] OR "pregnancies"[All Fields] OR "pregnancy s"[All Fields]) OR ("pregnant"[All Fields] OR "pregnants"[All Fields]) OR ("perinatal"[All Fields] OR "perinatally"[All Fields] OR "perinatals"[All Fields])) AND ("tanzania"[MeSH Terms] OR "tanzania"[All Fields] OR "tanzania s"[All Fields] OR "Tanga"[All Fields] OR "Arusha"[All Fields] OR "Dar es Salaam"[All Fields] OR "Dodoma"[All Fields] OR "Geita"[All Fields] OR "Iringa"[All Fields] OR "Kagera"[All Fields] OR "Katavi"[All Fields] OR "Kigoma"[All Fields] OR "Kilimanjaro"[All Fields] OR "Lindi"[All Fields] OR "Manyara"[All Fields] OR "Mara"[All Fields] OR "Mbeya"[All Fields] OR "Morogoro"[All Fields] OR "Mtwara"[All Fields] OR "Mwanza"[All Fields] OR "Njombe"[All Fields] OR (("indian ocean islands"[MeSH Terms] OR ("indian"[All Fields] AND "ocean"[All Fields] AND "islands"[All Fields]) OR "indian ocean islands"[All Fields] OR "pemba"[All Fields]) AND ("north"[All Fields] OR "norths"[All Fields])) OR (("indian ocean islands"[MeSH Terms] OR ("indian"[All Fields] AND "ocean"[All Fields] AND "islands"[All Fields]) OR "indian ocean islands"[All Fields] OR "pemba"[All Fields]) AND "south"[All Fields]) OR "Pwani"[All Fields] OR "Rukwa"[All Fields] OR "Ruvuma"[All Fields] OR "Shinyanga"[All Fields] OR "Simiyu"[All Fields] OR "Singida"[All Fields] OR "Tabora"[All Fields] OR "Tanga"[All Fields] OR (("tanzania"[MeSH Terms] OR "tanzania"[All Fields] OR "zanzibar"[All Fields]) AND ("north"[All Fields] OR "norths"[All Fields])) OR (("tanzania"[MeSH Terms] OR "tanzania"[All Fields] OR "zanzibar"[All Fields]) AND "south"[All Fields]) OR (("tanzania"[MeSH Terms] OR "tanzania"[All Fields] OR "zanzibar"[All Fields]) AND "west"[All Fields]))
